# DNA copy number variations and craniofacial abnormalities in 1,457 children with neurodevelopmental disorders

**DOI:** 10.1186/s13052-025-01839-6

**Published:** 2025-01-23

**Authors:** Dandan Wu, Ran Chen, Jerry Zhang, Wu Yan, Mengyin Chen, Dongqing Xia, Xiaonan Li, Yanyan Dai, Yinhua Chen, Rong Li

**Affiliations:** 1 Child Mental Health Deparment, Children’s Hospital of Nanjing Medical University, NanjingJiangsu, 210008 China; 2 Child Healthcare Department, Children’s Hospital of Nanjing Medical University, Jiangdong South No.8 Road, Nanjing, Jiangsu 210008 China; 3 Basis International School Nanjing, Nanjing, Jiangsu 210008 China

**Keywords:** Children, Copy-number variation, Microdeletions, Microduplications, Neurodevelopmental disorders, Whole-exome sequencing

## Abstract

**Background:**

This study aimed to investigate deoxyribonucleic acid (DNA) copy number variations (CNVs) in children with neurodevelopmental disorders and their association with craniofacial abnormalities.

**Methods:**

A total of 1,457 children who visited the Child Health Department of our hospital for unexplained Neurodevelopmental disorders (NDDs) between November 2019 and December 2022 were enrolled. Peripheral venous blood samples (2 mL) were collected from the children and their parents for whole-exome sequencing. Positive results were verified through Sanger sequencing for locus and pedigree validation. Simultaneously, a specific sign-scoring scale was created to evaluate characteristics related to the developments of eyes, nose, ears, eyebrows, head, mouth, face, trunk, limbs, and reproductive, urinary, and cardiovascular systems.

**Results:**

A total of 536 children (36.78%, 536/1,457) were found to have genetic variations, with 379 (70.71%, 379/536) exhibiting pathogenic monogenic mutations. Furthermore, 157 children (29.29%, 157/536) harbored DNA copy number variants, encompassing microdeletions (68.15%, 107/157) and microduplications (31.85%, 50/157). Regarding the pathogenicity of CNVs, 91 (57.96%, 91/157) were identified as pathogenic, 28 (17.83%, 28/157) as variants of uncertain clinical significance (VOUS), and 38 (24.20%, 38/157) as benign according to the American College of Medical Genetics and Genomics (ACMG).Using a specific sign-scoring scale, the proportion of pathogenic CNVs in children graded 1 point or higher (64%, 58/91) was significantly higher than that of non-pathogenic CNVs (43%, 29/66) (*P* < 0.05). Furthermore, the proportion of microdeletions in children graded 1 point or higher (60.75%, 65/107) was significantly higher than those carrying microduplications (44%, 22/50) (*P* < 0.05). The proportion of pathogenic microdeletions in children graded 1 point or higher (73.43%,47/64) was significantly higher than those carrying pathogenic microduplications (40.74%, 11/27) (*P* < 0.05).

**Conclusion:**

The positive rate of whole-exome sequencing for children with combined craniofacial abnormalities and NDDs exceeds the international average in our study cohort. Thus, whole-exome sequencing may be recommended for precise diagnosis of neurogenetic diseases in such cases.

**Supplementary Information:**

The online version contains supplementary material available at 10.1186/s13052-025-01839-6.

## Background

Neurodevelopmental disorders (NDDs) encompass a group of conditions that originate during the developmental stage. NDDs typically emerge in early life (often before school age), manifesting as developmental abnormalities that impair social, academic, or occupational functioning. NDDs include comprehensive developmental delay/intellectual disability (DD/ID) and autism spectrum disorders (ASD). Globally, DD/ID and ASD are closely associated with childhood disability. DD/ID, a group of neurodevelopmental disorders of high clinical and genetic heterogeneity, are characterized by ASD and attention deficit hyperactivity disorder. The prevalence of ID is around 1% worldwide, and that of severe ID is about 0.6% [1–3]. ASD has shown an escalating incidence since its initial recognition in 1943. The global prevalence of ASD is approximately 1%, according to the World Health Organization (WHO) data from 2012 [4].

The etiology of NDDs involves exogenous and genetic factors. Exogenous factors, like infections, toxins, trauma and malnutrition, can be well controlled [5]. Genetic factors pose an increasing weight on the pathogenesis of NDDs. For example, genetic factors are responsible for two-thirds of ID cases, including chromosomal abnormalities, single/multi-gene mutations, and congenital metabolic defects, which may result in developmental delay, mental disorders, distinctive facial features, endocrine abnormalities, and behavioral changes. Health education and rehabilitation can relieve clinical symptoms of ID patients, but pose a heavy financial cost [6–9].

DNA copy number variations (CNVs) are defined as alterations in deoxyribonucleic acid (DNA) stretches in contrast to a reference genome, with a size ranging from a kilobase to an entire chromosome (monosomy/trisomy). CNVs, which may appear in multiple, single or intergenic regions, are pathogenic or benign [10]. Chromosomal microdeletions and microduplications constitute a portion of CNVs, but either microdeletions or microduplications, though emerging in the same region, may lead to inconsistent phenotypes, bringing patients with clinical and genetic heterogeneities. New genetic technology has made possible to deepen into the etiology in NDDs, including molecular analysis (a-CGH) and NGS (panels, whole-exome sequencing, whole-genome sequencing).

Children with NDDs may display craniofacial and organ malformations alongside brain dysfunction. Craniofacial malformations are related to the abnormal growth of cranial neural crest cells (CNCCs) caused by genetic variants, such as choromosomal deletions or duplications [11]. Unique facial features are common in numerous syndromes, and seldom change with time. Some genetic diseases are often recognized by distinctive facial features; however, their rarity remains a challenge for clinicians, who have to identify an ever-growing number of different genetic disorders. 3D imaging offers preliminary facial screening, but its application is limited also due to the high costs. For NDDs children displaying craniofacial malformations, a new tool method may be useful for clinical diagnosis and analysis.

This study aimed to identify DNA CNVs though whole-exome sequencingin a group of children with DD/ID or ASD. We also designed a new diagnostic scale to assess craniofacial features and analyze children’s clinical phenotypes. We examined the correlation between DNA CNVs and clinical phenotypes. Therefore, an assessment of special facial characteristics using the sign-scoring scale favored the early screening and recognition of high-risk genetic diseases, as well as whole-exome or whole-genome sequencing for a pedigree study. A timely intervention by family members and professional institutions would greatly benefit children with NDDs, thus reducing family and social burdens and improving the quality of life of the affected subjects.

## Methods

### Participants

A total of 1,457 children diagnosed with unexplained intellectual disability (ID) were included. These children were treated at the Child Health Department of the Children’s Hospital of Nanjing Medical University (Nanjing, China) from November 2019 to December 2022.

Inclusion criteria: (1) Children under 4 years old were assessed using Gesell and Griffiths diagnostic scales. If two or more developmental areas (including adaptability, large movement, fine movement, language, and personal social/behavior) differed by more than two standard deviations, comprehensive developmental delay was considered. Children scoring below 50 points in five developmental areas were included. (2) Intelligence Quotient (IQ) was measured using Wechsler scales in children aged 4–14. Based on IQ value and social adaptability, ID was categorized as mild (IQ: 50–69), moderate (IQ: 35–49), severe (IQ: 20–34), or extremely severe (IQ: < 20). All enrolled children had moderate, severe, or extremely severe ID. (3) Autism spectrum disorders (ASD) were diagnosed according to the criteria outlined in the US Diagnostic and Statistical Manual of Mental Disorders, Fifth Edition (DSM-5).

Excluded were those with (1) known specific genetic diseases or genetic metabolic diseases indicated by clinical manifestations and laboratory examinations; (2) DD/ID or ASD caused by perinatal ischemia, hypoxic brain injury, kernicterus, central nervous system infection, poisoning, or a history of trauma [12].

### Procedures

With the informed consent from children’s guardians, 2 ml of peripheral venous blood was collected from each of the child and his or her parents for whole-exome sequencing and CNV analysis. For those with positive results, mutation site and pedigree were confirmed through the Sanger sequencing.

Target Region Capture Sequencing: the DNA fragments within the target region were enriched and subsequently sequenced using a high-throughputsecond-generation sequencing platform. In this study, a total of 23 exonic regions in 23,000 genes were captured using MyGenostics’ GenCap kit through procedures of randomly fragmenting genomic DNA, ligating it to the Illumina (NovaSeq6000, USA) PE adaptor oligonucleotide mixture, and performing link-mediated polymerase chain reaction (ligation-mediated PCR) amplification and purification to create the DNA library. Quality testing was conducted, and PCR products were hybridized into a target region capture chip to enrich the sequences of interest. Subsequently, the enriched sequences were sequenced using the Illumina Nova 6000 sequencer, followed by preliminary raw data processing, including image recognition and sample differentiation.

Bioinformatic Analysis: contaminated and overlapped data were removed. Then, utilizing BWA software (http://bio-bwa.sourceforge.net/) [13, 14], the filtered sequences were aligned against the human genome reference sequence (hg19) from the NCBI database. GATK software (https://software.broadinstitute.org/gatk/) [15] was employed to analyze and identify single nucleotide variants (SNVs) and insertion-deletion mutations (INDELs). A further annotation of all single nucleotide polymorphisms (SNPs) and INDELs were annotated using ANNOVAR software (http://annovar.openbioinformatics.org/en/latest/) [16]. Mutation sites at a frequency lower than 0.05 were selected from the normal human databases, including the Thousand Genomes Project (http://www.1000genomes.org), Exome Variant Server (http://evs.gs.washington.edu/EVS) and EXAC (http://exac.broadinstitute.org/). Pathogenicity and conservative predictions were performed on SIFT (http://sift.jcvi.org/) [17–19], PolyPhen-2 (http://genetics.bwh.harvard.edu/pph2/) [20–22], MutationTaster (http://www.mutationtaster.org/) [23], and GERP + + (http://mendel.stanford.edu/SidowLab/downloads/gerp/index.html) [24]. Splicing sites were analyzed with SPIDEX (http://www.deepgenomics.com/spidex) software.

PCR Amplification and Sanger Sequencing: variant sites were further verified via PCR amplification and Sanger sequencing. The PCR primer pairs were designed using the online Primer 3.0 software [25–27]. Subsequently, the PCR products were subjected to Sanger sequencing and analyzed on an ABI 3130 Genetic Analyzer (Applied Biosystems Genetic Analyzer, 3130, USA). Family members were assessed for co-segregation.

We have designed a rating scale for craniofacial anomalies in children with neurodevelopmental disorders in the Procedures section (Table 1). Based on the assessment of craniofacial anomalies specifically in the head, skin/hair, face, eyebrows, eyes, ears, nose, oral cavity and other parts, any anomaly (single or multiple anomalies) in one craniofacial region was graded as 1 point, otherwise it was graded as 0. For example, a 4-year-old boy with positive characteristics of wide eye fissure, bilateral bulge, too long eyelash, low nasal bridge and asymmetric nostrils was graded as 2 points, including 1 point for three anomalies in the eyes and 1 for two anomalies in the nose.

**Table 1:** A self-designed rating scale for craniofacial anomalies in children with neurodevelopmental disorders

Craniofacial region	Positive characteristics
Head	Too big/small, abnormal head shape
Skin/Hair	Dark/fair/marbled skin, Café au lait spots, rash, yellow/white hair, thin hair, low hairline, capillary nevus
Face	Small mandible, mandibular lordosis/retroversion, temporal depression, elongated cheek, triangular/flat face, facial asymmetry, medium long/convex, convex/wide/narrow forehead
Eyebrows	Bushy/even/arched/too long/no/sparce eyebrows
Eyes	Narrow/wide eye fissure, almond eye, wide eye distance, superior oblique angle of outer canthus, epicanthus, inferior oblique eyelid fissure, strabismus, eye cohesion, nystagmus, iris abnormality, scleral color abnormality, eye socket edema, bilateral depression, bilateral bulge, eyelid droop, too long/dense/missing eyelash
Ears	Low ear position, small/large external ear, pinna abnormalities (pointed ear, windy ear, simple pinna, shape deformity), large/attached earlobe
Nose	Low/high nasal bridge, wide/concave nasal root, round nose, asymmetric/upward sloping/large nostrils
Oral cavity	Cleft palate, cleft/thick lip, cracked tongue, abnormal gums, abnormal tooth development, large tongue, tongue extension, abnormal tongue shape, narrow hard palate, raised/thin upper lip, small mouth, drooping corners of the mouth, mouth slanting, high palate arch
Other parts	Trunk/limbs, congenital heart disease, urinary malformations (cryptorchidism, hypospadias, small penis, large testis, abnormal labial development)

### Statistical analysis

Data were recorded using EpiData 3.0, and SPSS 22.0 software was used for statistical analysis. Numeration data were expressed in percentage or frequency, and compared using the Chi-square test. *P* < 0.05 indicated statistical significance.

## Results

### General situation analysis

Out of the 1,457 children, 536 (36.78%) were identified to carry genetic variations. Among them, 379 (70.71%) exhibited pathogenic single-gene mutations. Additionally, 157 children (29.29%) presented DNA CNVs, including 107 (68.15%) with microdeletion and 50 (31.85%) with microduplication. Among the 157 children with CNVs, 85 were male and 72 were female. Their ages ranged from 1 month to 13 years, with a mean age of 3.98 ± 2.68 years (Table 2).

**Table 2:** Basic characteristics of NDD patients

Characteristics	Number of patients (%)
Total	157
Sex	
Male	85(54.14%, 85/157)
Female	72(45.85%,72/157)
Age	
≤ 1 year	55(35.03%, 56/157)
> 1 year, ≤ 3 years	55(35.03%, 55/157)
> 3 year, ≤ 6 years	23(14.65%, 23/157)
> 6 year	24(15.29%, 24/157)
Genetic etiology	
Microdeletion	107 (68.15%, 107/157)
Microduplication	50 (31.85%, 50/157)

### Pathogenicity classification of CNVs

Of the 157 CNVs, 91 (57.96%, 91/157) were pathogenic, 28 (17.83%, 28/157) benign, and 38 (24.20%, 38/157) VOUS [28, 29] (Table S1).

**Fig. 1:**
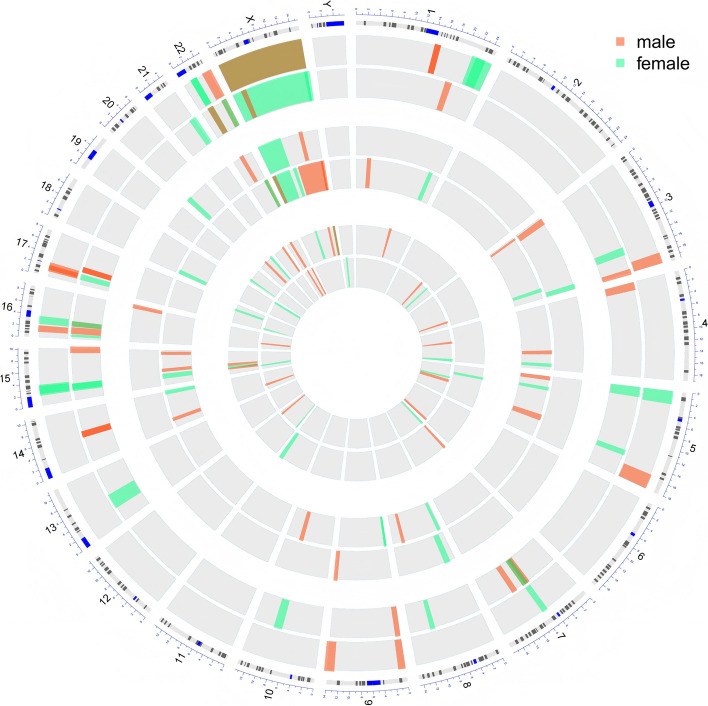
Distribution of pathogenic CNVs on chromosomes (1. red means male and green means female; 2. Pathogenic/ Likely pathogenic/ Uncertain significance from outside to inside; 3. A direction toward the center of the circle indicates deletion, and outward indicates duplication.)

Among the 91 pathogenic CNVs, 20 (21.98%, 20/91) were located at chromosome 7, 13 (14.29%, 13/91) at chromosome X, 11 (12.09%, 11/91) at chromosome 15. Williams syndrome, observed in 16 cases (17.58%, 16/91), was the most common (Fig. 1).

### Analysis of clinical special signs of DNA CNVs

CNVs were divided into six groups for analysis of clinical special signs: microdeletion group, microduplication group, pathogenic group, non-pathogenic group (benign CNVs and VOUS), pathogenic microdeletion group and pathogenic microduplication group.

In the microdeletion group, 65 cases (60.75%) were scored by ≥ 1 point, significantly more than 22 cases (44%) in the microduplication group (*P* < 0.05). In the pathogenic CNVs group, 58 cases (64%) were scored by ≥ 1 point, significantly more than the 29 cases (43%) in the non-pathogenic CNVs group (*P* < 0.05). In the pathogenic microdeletion group, 47 cases (73.43%) were scored by ≥ 1 point, significantly more than the 11 cases (40.74%) in the pathogenic microduplication group (Table 3). The probabilities of eye deformity in 32 cases (35%) and oral deformity in 38 cases (42%) were higher in the pathogenic case group, and oral deformity was more likely to occur in either the microdeletion group and the microduplication group or the pathogenic microdeletion group and the pathogenic microduplication group (*P* < 0.05) (Table 4).

**Table 3:** Special sign scale scores in six groups of different DNA CNVs

Group	Special Signs Scale		*χ*^*2*^	*P*
	0 points	> = 1 point		
Microdeletion group (*N* = 107)	42(39.25%, 42/107)	65(60.75%, 65/107)	3.869	0.049
Microduplication group (*N* = 50)	28(56%, 28/50)	22(44%, 22/50)		
Pathogenic group (*N* = 91)	33(36.26%, 33/91)	58(63.74%, 58/91)	7.014	0.008
Non-pathogenic group (*N* = 66)	38(56.72%, 38/67)	28(41.79%, 28/67)		
Pathogenic microdeletion group (*N* = 64)	17(26.56%, 17/64)	47(73.43%, 47/64)	8.783	0.003
Pathogenic microduplication group (*N* = 27)	16(59.26%, 16/27)	11(40.74%, 11/27)

**Table 4:** Comparison of cranial and facial signs among six groups of CNVs

Group	Facial parts							
	Head	Skin/hair	Face	Eyebrow	Eye	Ear	Nose	Oral cavity
Pathogenic group (*N* = 91)	21 (24%)	5 (5%)	14 (15%)	4 (4%)	32 (35%)	13 (14%)	7 (8%)	38 (42%)
Non-pathogenic group (*N* = 66)	12 (18%)	2 (3%)	4 (6%)	7 (11%)	9 (14%)	7 (11%)	4 (6%)	13 (20%)
*χ*^*2*^	0.552	0.545	3.276	2.265	9.189	0.466	0.156	8.489
*P*	0.457	0.46	0.07	0.132	0.002	0.495	0.693	0.004
Microdeletion group (*N *= 107)	25 (23%)	6 (6%)	4 (4%)	8 (8%)	30 (28%)	14 (13%)	8 (11%)	34 (32%)
Microduplication group (*N* = 50)	8 (16%)	1 (2%)	3 (6%)	3 (6%)	10 (20%)	6 (12%)	3 (6%)	7 (14%)
*χ*^*2*^	1.113	1.041	0.409	0.114	1.159	0.036	0.114	5.580
*P*	0.291	0.308	0.522	0.736	0.282	0.849	0.736	0.018
Pathogenic microdeletion group (*N* = 64)	18 (28%)	4 (6%)	10 (16%)	1 (2%)	25 (39%)	9 (14%)	6 (9%)	36 (56%)
Pathogenic microduplication group (*N* = 27)	3 (11%)	1 (4%)	4 (15%)	3 (11%)	7 (26%)	4 (15%)	1 (4%)	2 (7%)
*χ*^***2***^	3.097	0.237	0.010	3.679	1.437	0.009	0.86	18.626
*P*	0.078	0.606	0.922	0.055	0.231	0.925	0.354	0.000

## Discussion

NDDs, presenting different manifestations, often bring a high rate of disability, but a lack of effective treatment options. Genetic factors play a pivotal role in their etiology, spanning chromosomal abnormalities, single or multigene mutations, and congenital metabolic defects. Notably, children with the same NDD exhibit similar craniofacial and other organ malformations, implying that common genetic factors may underpin craniofacial malformations.

Craniofacial development occurs during the gastrula stage, and needs the orchestration of diverse signaling pathways and germ layers to ensure a normal morphogenesis. Cranial neural crest cells (CNCCs) are key contributors to craniofacial development. Although craniofacial traits exhibit a strong heritability, however, a high heterogeneity exists in different population and individuals [30]. Genome-wide association analysis have identified over 300 *loci* linked to facial morphogenesis, predominantly within the enhancer regions of neural crest cells or fetal maxillofacial tissues [31].

Genetic mutations are the culprit of congenital craniofacial malformations [32], such as head shape anomalies, facial irregularities (e.g., low nasal bridge, asymmetrical eyes, sparse eyelashes, ear dysplasia), hair aberrations, and other features. [33–35]. Numerous studies have associated specific genes with various phenotypic facial features, such as teeth, ears, and hair [36, 37]. Growing evidence within the past decade has supported the enhanced accuracy of next-generation sequencing (NGS) in diagnosing NDDs [38]. NGS is the preferred examination in children with NDDs, although a systematic guideline for its clinical application is scant [39–46]. Whole-exome sequencing offers a higher diagnostic rate of NDDs than chromosomal microarray analysis (CMA) and array comparative genomic hybridization (aCGH) [45, 46], and is recommended as a first-tier diagnostic test. In our center, we also performed whole-exome sequencing to identify children with NDDs. Besides, according to these associations and our clinical experience, we established a special sign scale to evaluate these deformities.

In the present study, we carried out genetic testing for NDD children exhibiting a special sign score of ≥ 1. Among the 1,457 children, 536 cases were diagnosed with genetic variations, achieving a diagnostic rate of 36.78%. The rate of single pathogenic CNV was 25.88%, and that of DNA copy number variation was 10.78%, significantly higher than earlier reported 15%−20% [47–49]. The scale improved the positive diagnostic rate in outpatients, demonstrating its discriminative value.

CNVs constitute approximately 13% of the genome [50], and a considerable portion of them are benign [51]. However, pathogenic CNVs may lead to psychiatric and neurodevelopmental disorders, somatic diseases, neurological conditions, and cancers [52]. However, there lack specific tools in diagnosing pathogenic CNVs. The present study revealed that the likelihood of pathogenic CNVs was notably higher in children with a score of ≥ 1 by the special sign scale, suggesting that the scale holds huge potential to predict the pathogenicity of CNVs.

DNA alterations, including CNVs, result from the natural evolution and adaptation of organisms [52]. In humans, deletions and duplications are more balanced than in other mammals [53]. Some repeats play a crucial role in driving human evolution, leading to brain enlargement and the emergence of novel human-specific genes [54]. In theory, deletion and duplication should happen simultaneously with the same frequencies, but research has shown that CNVs are more likely to occur during early gamete formation. Clinical observations have also highlighted that microduplications are less deleterious and may lack clinical phenotypes. Moreover, at the chromosome level, the tolerance of an individual to triploidy is higher than that to haploidy, implying that triploids have a higher survival rate than haploids [55–57]. Our study further revealed that children in the deletion group displayed more craniofacial abnormalities, particularly oral malformations, in the special sign scale. These findings underscore that gene deletions tend to be more detrimental than duplications [58, 59]. Interestingly, our research identified 7q11.23 deletions as the most frequent cause of Williams syndrome, a finding that contrasts with previous studies on microdeletions and microduplications. As such, additional clinical evidence is required to ascertain the effect of deletions and duplications on human development. We identified that certain genes, such as *GTF2I*, *LAT 2*, *LIMK 1*, *ARSA*, and *SHANK3*, were associated with higher scores on the assessment scale. However, the extent to which these genes correlate with CNCC development warrants further investigation.

In the present study, 157 children harbored DNA CNVs, including 107 (68%) with microdeletions and 50 (32%) with microduplications. Among these, 91 cases (58%) were unequivocally pathogenic. Notably, most of the pathogenic CNVs were located on chromosome 7 (20%), followed by X chromosome (14%) and chromosome 15 (12%). These findings deviate from prior studies that reported chromosomes 18 and 22 as the most common sources of pathogenic CNVs [60]. These differences may be attributed to sample size and age. Thus, further exploration is warranted through studies involving a larger sample of children with neurodevelopmental disorders.

Overall, we created a specific sign-scoring scale to evaluate craniofacial abnormalities associated with NDDs and provided more references for illustrating the phenotype-genotype relationship of NDDs. In addition,craniofacial abnormalities indicated by the sign-scoring scale, especially anomalies in the eyes and oral cavity, significantly improve the positive rate of whole-exome sequencing. The use of the sign-scoring scale is conductive to the increased etiological diagnosis rate of genetic diseases and rare diseases, allowing for a more effective allocation of medical resources.We think broadly about the future use of the sign-scoring scale to neonatal screening, especially in parents with abnormal family and gestational history. The sign-scoring scale was able to provide an alarm for high-risk populations to rapidly receive genetic counseling, whole-exome sequencing and interventions.

### Limitations

Several limitations warrant consideration in this study. Firstly, the sample was mainly pooled from Nanjing and surrounding areas. Secondly, all subjects are Han Chinese, which may bring with ethnicity-related bias. Consequently, the study results may not be representative for the overall Chinese population.

## Conclusions

Craniofacial abnormalities represent significant clinical phenotypes associated with DNA copy number variations in children. The special sign scale, primarily focusing on cranial and facial anomalies, can be used to guide primary screening and genetic testing for children with NDDs.

## Supplementary Information

Supplementary Material 1.

## Data Availability

The data used to support the findings of this study are available from the corresponding author upon request.

## Abbreviations

DNA: Deoxyribonucleic acid
CNVs: Copy number variations
NDDs: Neurodevelopmental disorders
DD/ID: Developmental delay/intellectual disability
ASD: Autism spectrum disorders
WHO: World Health Organization
CNCCs: Cranial neural crest cells
IQ: Intelligence Quotient
PCR: Polymerase chain reaction
SNVs: Single nucleotide variants
INDELs: Insertion-deletion mutations
SNPs: Single nucleotide polymorphisms

## References

[1] Friedman C. Outdated language: use of “mental retardation” in medicaid HCBS waivers post-Rosa’s Law. Intellect Dev Disabil. 2016;54:342–53. 10.1352/1934-9556-54.5.342. 27673735 10.1352/1934-9556-54.5.342

[2] Maenner MJ, Blumberg SJ, Kogan MD, Christensen D, Yeargin-Allsopp M, Schieve LA. Prevalence of cerebral palsy and intellectual disability among children identified in two U.S. national surveys, 2011–2013. Ann Epidemiol. 2016;26:222–6. 10.1016/j.annepidem.2016.01.001. 26851824 10.1016/j.annepidem.2016.01.001 PMC5144825

[3] Maulik PK, Mascarenhas MN, Mathers CD, Dua T, Saxena S. Prevalence of intellectual disability: a meta-analysis of population-based studies [published correction appears in Res Dev Disabil. 2013 Feb;34(2):729]. Res Dev Disabil. 2011;32(2):419–436. 10.1016/j.ridd.2010.12.018. 10.1016/j.ridd.2010.12.018 21236634

[4] World Health Organization. Meeting report: autism spectrum disorders and other developmental disorders: from raising awareness to building capacity. Geneva: World Health Organization; 2013.

[5] Piro E, Serra G, Schierz IAM, Giuffrè M, Corsello G. Neonatal ten-year retrospective study on neural tube defects in a second level University Hospital. Ital J Pediatr. 2020;46(1):72. 10.1186/s13052-020-00836-1. Published 2020 May 24. 32448340 10.1186/s13052-020-00836-1 PMC7247239

[6] Ma Y, Chen C, Wang Y, et al. Analysis copy number variation of Chinese children in early-onset epileptic encephalopathies with unknown cause. Clin Genet. 2016;90(5):428–36. 10.1111/cge.12768. 26925868 10.1111/cge.12768

[7] Vissers LE, Gilissen C, Veltman JA. Genetic studies in intellectual disability and related disorders. Nat Rev Genet. 2016;17(1):9–18. 10.1038/nrg3999. 26503795 10.1038/nrg3999

[8] Chiurazzi P, Pirozzi F. Advances in understanding - genetic basis of intellectual disability. F1000Res. 2016;5:F1000 Faculty Rev-599. 10.12688/f1000research.7134.1. Published 2016 Apr 7. 27127621 10.12688/f1000research.7134.1 PMC4830215

[9] O’Byrne JJ, Lynch SA, Treacy EP, et al. Unexplained developmental delay/learning disability: guidelines for best practice protocol for first line assessment and genetic/metabolic/radiological investigations. Ir J Med Sci. 2016;185(1):241–8. 10.1007/s11845-015-1284-7. 25894277 10.1007/s11845-015-1284-7

[10] Watson CT, Marques-Bonet T, Sharp AJ, Mefford HC. The genetics of microdeletion and microduplication syndromes: an update. Annu Rev Genomics Hum Genet. 2014;15:215–44. 10.1146/annurev-genom-091212-153408. 24773319 10.1146/annurev-genom-091212-153408 PMC4476258

[11] Siismets EM, Hatch NE. Cranial neural crest cells and their role in the pathogenesis of craniofacial anomalies and coronal craniosynostosis. J Dev Biol. 2020;8(3):18. 10.3390/jdb8030018. Published 2020 Sep 9. 32916911 10.3390/jdb8030018 PMC7558351

[12] Piro E, Serra G, Schierz IAM, Giuffrè M, Corsello G. Fetal growth restriction: a growth pattern with fetal, neonatal and long-term consequences. Euromediterranean Biomed J. 2019;14(09):038–44.

[13] Li H, Durbin R. Fast and accurate short read alignment with Burrows-Wheeler transform. Bioinformatics. 2009;25(14):1754–60. 10.1093/bioinformatics/btp324. 19451168 10.1093/bioinformatics/btp324 PMC2705234

[14] Li H, Durbin R. Fast and accurate long-read alignment with Burrows-Wheeler transform. Bioinformatics. 2010;26(5):589–95. 10.1093/bioinformatics/btp698. 20080505 10.1093/bioinformatics/btp698 PMC2828108

[15] McKenna A, Hanna M, Banks E, et al. The genome analysis Toolkit: a MapReduce framework for analyzing next-generation DNA sequencing data. Genome Res. 2010;20(9):1297–303. 10.1101/gr.107524.110. 20644199 10.1101/gr.107524.110 PMC2928508

[16] Wang K, Li M, Hakonarson H. ANNOVAR: functional annotation of genetic variants from high-throughput sequencing data. Nucleic Acids Res. 2010;38(16):e164. 10.1093/nar/gkq603. 20601685 10.1093/nar/gkq603 PMC2938201

[17] Choi Y, Sims GE, Murphy S, Miller JR, Chan AP. Predicting the functional effect of amino acid substitutions and indels. PLoS ONE. 2012;7(10):e46688. 10.1371/journal.pone.0046688. 23056405 10.1371/journal.pone.0046688 PMC3466303

[18] Choi Y . A fast computation of pairwise sequence alignment scores between a protein and a set of single-locus variants of another protein[C]. Acm Conference on Bioinformatics. ACM, 2012. 10.1145/2382936.2382989.

[19] Choi Y, Chan AP. PROVEAN web server: a tool to predict the functional effect of amino acid substitutions and indels. Bioinformatics. 2015;31(16):2745–7. 10.1093/bioinformatics/btv195. 25851949 10.1093/bioinformatics/btv195 PMC4528627

[20] Adzhubei I, Jordan DM, Sunyaev SR. Predicting functional effect of human missense mutations using PolyPhen-2. Curr Protoc Hum Genet. 2013;Chapter 7:Unit7.202undefined0undefined. 10.1002/0471142905.hg0720s76. 10.1002/0471142905.hg0720s76 PMC4480630 23315928

[21] Ramensky V, Bork P, Sunyaev S. Human non-synonymous SNPs: server and survey. Nucleic Acids Res. 2002;30(17):3894–900. 10.1093/nar/gkf493. 12202775 10.1093/nar/gkf493 PMC137415

[22] Sunyaev SR, Eisenhaber F, Rodchenkov IV, Eisenhaber B, Tumanyan VG, Kuznetsov EN. PSIC: profile extraction from sequence alignments with position-specific counts of independent observations. Protein Eng. 1999;12(5):387–94. 10.1093/protein/12.5.387. 10360979 10.1093/protein/12.5.387

[23] Schwarz JM, Cooper DN, Schuelke M, Seelow D. MutationTaster2: mutation prediction for the deep-sequencing age. Nat Methods. 2014;11(4):361–2. 10.1038/nmeth.2890. 24681721 10.1038/nmeth.2890

[24] Untergasser A, Cutcutache I, Koressaar T, et al. Primer3–new capabilities and interfaces. Nucleic Acids Res. 2012;40(15):e115. 10.1093/nar/gks596. 22730293 10.1093/nar/gks596 PMC3424584

[25] Koressaar T, Remm M. Enhancements and modifications of primer design program Primer3. Bioinformatics. 2007;23(10):1289–91. 10.1093/bioinformatics/btm091. 17379693 10.1093/bioinformatics/btm091

[26] Kõressaar T, Lepamets M, Kaplinski L, Raime K, Andreson R, Remm M. Primer3_masker: integrating masking of template sequence with primer design software. Bioinformatics. 2018;34(11):1937–8. 10.1093/bioinformatics/bty036. 29360956 10.1093/bioinformatics/bty036

[27] Cooper GM, Stone EA, Asimenos G, et al. Distribution and intensity of constraint in mammalian genomic sequence. Genome Res. 2005;15(7):901–13. 10.1101/gr.3577405. 15965027 10.1101/gr.3577405 PMC1172034

[28] Kearney HM, Thorland EC, Brown KK, Quintero-Rivera F, South ST, Working Group of the American College of Medical Genetics Laboratory Quality Assurance Committee. American College of Medical Genetics standards and guidelines for interpretation and reporting of postnatal constitutional copy number variants. Genet Med. 2011;13(7):680–5. 10.1097/GIM.0b013e3182217a3a. 21681106 10.1097/GIM.0b013e3182217a3a

[29] Riggs ER, Andersen EF, Cherry AM, et al. Technical standards for the interpretation and reporting of constitutional copy-number variants: a joint consensus recommendation of the American College of Medical Genetics and Genomics (ACMG) and the Clinical Genome Resource (ClinGen) [published correction appears in Genet Med. 2021;23(11):2230. 10.1038/s41436-021-01150-9 33731880

[30] White JD, Indencleef K, Naqvi S, et al. Insights into the genetic architecture of the human face. Nat Genet. 2021;53(1):45–53. 10.1038/s41588-020-00741-7. 33288918 10.1038/s41588-020-00741-7 PMC7796995

[31] Chang M, He L, Cai L. An overview of genome-wide association studies. Methods Mol Biol. 2018;1754:97–108. 10.1007/978-1-4939-7717-8_6. 29536439 10.1007/978-1-4939-7717-8_6

[32] Schmetz A, Amiel J, Wieczorek D. Genetics of craniofacial malformations. Semin Fetal Neonatal Med. 2021;26(6):101290. 10.1016/j.siny.2021.101290. 34561177 10.1016/j.siny.2021.101290

[33] Park JH, Yamaguchi T, Watanabe C, et al. Effects of an Asian-specific nonsynonymous EDAR variant on multiple dental traits. J Hum Genet. 2012;57(8):508–14. 10.1038/jhg.2012.60. 22648185 10.1038/jhg.2012.60

[34] Adhikari K, Reales G, Smith AJ, et al. A genome-wide association study identifies multiple loci for variation in human ear morphology. Nat Commun. 2015;6:7500. 10.1038/ncomms8500. Published 2015 Jun 24. 26105758 10.1038/ncomms8500 PMC4491814

[35] Morgan MD, Pairo-Castineira E, Rawlik K, et al. Genome-wide study of hair colour in UK Biobank explains most of the SNP heritability. Nat Commun. 2018;9(1):5271. 10.1038/s41467-018-07691-z. Published 2018 Dec 10. 30531825 10.1038/s41467-018-07691-z PMC6288091

[36] Claes P, Roosenboom J, White JD, et al. Genome-wide mapping of global-to-local genetic effects on human facial shape. Nat Genet. 2018;50(3):414–23. 10.1038/s41588-018-0057-4. 29459680 10.1038/s41588-018-0057-4 PMC5937280

[37] White JD, Ortega-Castrillón A, Matthews H, et al. MeshMonk: open-source large-scale intensive 3D phenotyping. Sci Rep. 2019;9(1):6085. 10.1038/s41598-019-42533-y. Published 2019 Apr 15. 30988365 10.1038/s41598-019-42533-y PMC6465282

[38] Schierz IAM, Serra G, Antona V, Persico I, Corsello G, Piro E. Infant developmental profile of Crisponi syndrome due to compound heterozygosity for CRLF1 deletion. Clin Dysmorphol. 2020;29(3):141–3. 10.1097/MCD.0000000000000325.. 32433043 10.1097/MCD.0000000000000325

[39] Thevenon J, Duffourd Y, Masurel-Paulet A, et al. Diagnostic odyssey in severe neurodevelopmental disorders: toward clinical whole-exome sequencing as a first-line diagnostic test. Clin Genet. 2016;89(6):700–7. 10.1111/cge.12732. 26757139 10.1111/cge.12732

[40] Clark MM, Stark Z, Farnaes L, et al. Meta-analysis of the diagnostic and clinical utility of genome and exome sequencing and chromosomal microarray in children with suspected genetic diseases. NPJ Genom Med. 2018;3:16. 10.1038/s41525-018-0053-8. Published 2018 Jul 9. 30002876 10.1038/s41525-018-0053-8 PMC6037748

[41] Lindstrand A, Eisfeldt J, Pettersson M, et al. From cytogenetics to cytogenomics: whole-genome sequencing as a first-line test comprehensively captures the diverse spectrum of disease-causing genetic variation underlying intellectual disability. Genome Med. 2019;11(1):68. 10.1186/s13073-019-0675-1. Published 2019 Nov 7. 31694722 10.1186/s13073-019-0675-1 PMC6836550

[42] van der Sanden BPGH, Schobers G, Corominas Galbany J, et al. The performance of genome sequencing as a first-tier test for neurodevelopmental disorders. Eur J Hum Genet. 2023;31(1):81–8. 10.1038/s41431-022-01185-9. 36114283 10.1038/s41431-022-01185-9 PMC9822884

[43] Alotibi RS, Sannan NS, AlEissa M, et al. The diagnostic yield of CGH and WES in neurodevelopmental disorders. Front Pediatr. 2023;11:1133789. 10.3389/fped.2023.1133789. Published 2023 Mar 1. 36937954 10.3389/fped.2023.1133789 PMC10014736

[44] Srivastava S, Love-Nichols JA, Dies KA, et al. Meta-analysis and multidisciplinary consensus statement: exome sequencing is a first-tier clinical diagnostic test for individuals with neurodevelopmental disorders [published correction appears in Genet Med. 2020 Oct;22(10):1731–1732]. Genet Med. 2019;21(11):2413–21. 10.1038/s41436-019-0554-6. 31182824 10.1038/s41436-019-0554-6 PMC6831729

[45] Martinez-Granero F, Blanco-Kelly F, Sanchez-Jimeno C, et al. Comparison of the diagnostic yield of aCGH and genome-wide sequencing across different neurodevelopmental disorders. NPJ Genom Med. 2021;6(1):25. 10.1038/s41525-021-00188-7. Published 2021 Mar 25. 33767182 10.1038/s41525-021-00188-7 PMC7994713

[46] Serra G, Antona V, Giuffrè M, et al. Interstitial deletions of chromosome 1p: novel 1p31.3p22.2 microdeletion in a newborn with craniosynostosis, coloboma and cleft palate, and review of the genomic and phenotypic profiles. Ital J Pediatr. 2022;48(1):38. 10.1186/s13052-022-01232-7. Published 2022 Mar 4. 35246213 10.1186/s13052-022-01232-7 PMC8896361

[47] Roselló M, Martínez F, Monfort S, Mayo S, Oltra S, Orellana C. Phenotype profiling of patients with intellectual disability and copy number variations. Eur J Paediatr Neurol. 2014;18(5):558–66. 10.1016/j.ejpn.2014.04.010. 24815074 10.1016/j.ejpn.2014.04.010

[48] Pfundt R, Kwiatkowski K, Roter A, et al. Clinical performance of the CytoScan Dx assay in diagnosing developmental delay/intellectual disability. Genet Med. 2016;18(2):168–73. 10.1038/gim.2015.51. 25880438 10.1038/gim.2015.51

[49] Kashevarova AA, Nazarenko LP, Skryabin NA, et al. Array CGH analysis of a cohort of Russian patients with intellectual disability. Gene. 2014;536(1):145–50. 10.1016/j.gene.2013.11.029. 24291026 10.1016/j.gene.2013.11.029

[50] Stankiewicz P, Lupski JR. Structural variation in the human genome and its role in disease. Annu Rev Med. 2010;61:437–55. 10.1146/annurev-med-100708-204735. 20059347 10.1146/annurev-med-100708-204735

[51] Iafrate AJ, Feuk L, Rivera MN, et al. Detection of large-scale variation in the human genome. Nat Genet. 2004;36(9):949–51. 10.1038/ng1416. 15286789 10.1038/ng1416

[52] Hastings PJ, Lupski JR, Rosenberg SM, Ira G. Mechanisms of change in gene copy number. Nat Rev Genet. 2009;10(8):551–64. 10.1038/nrg2593. 19597530 10.1038/nrg2593 PMC2864001

[53] Hahn MW, Demuth JP, Han SG. Accelerated rate of gene gain and loss in primates. Genetics. 2007;177(3):1941–9. 10.1534/genetics.107.080077. 17947411 10.1534/genetics.107.080077 PMC2147951

[54] Dennis MY, Eichler EE. Human adaptation and evolution by segmental duplication. Curr Opin Genet Dev. 2016;41:44–52. 10.1016/j.gde.2016.08.001. 27584858 10.1016/j.gde.2016.08.001 PMC5161654

[55] Panigrahi I, Jain P, Didel S, et al. Identification of microdeletion and microduplication syndromes by chromosomal microarray in patients with intellectual disability with dysmorphism. Neurol India. 2018;66(5):1370–6. 10.4103/0028-3886.241346. 30233006 10.4103/0028-3886.241346

[56] Mostovoy Y, Yilmaz F, Chow SK, et al. Genomic regions associated with microdeletion/microduplication syndromes exhibit extreme diversity of structural variation. Genetics. 2021;217(2):iyaa038. 10.1093/genetics/iyaa038. 33724415 10.1093/genetics/iyaa038 PMC8045732

[57] Mohammadzadeh A, Akbaroghli S, Aghaei-Moghadam E, et al. Investigation of chromosomal abnormalities and microdeletion/ microduplication(s) in fifty Iranian patients with multiple congenital anomalies. Cell J. 2019;21(3):337–49. 10.22074/cellj.2019.6053. 31210441 10.22074/cellj.2019.6053 PMC6582423

[58] Emerson JJ, Cardoso-Moreira M, Borevitz JO, Long M. Natural selection shapes genome-wide patterns of copy-number polymorphism in Drosophila melanogaster. Science. 2008;320(5883):1629–31. 10.1126/science.1158078. 18535209 10.1126/science.1158078

[59] Cooper GM, Coe BP, Girirajan S, et al. A copy number variation morbidity map of developmental delay [published correction appears in Nat Genet. 2014 Sep;46(9):1040]. Nat Genet. 2011;43(9):838–46. 10.1038/ng.909. Published 2011 Aug 14. 21841781 10.1038/ng.909 PMC3171215

[60] Chaves TF, Baretto N, Oliveira LF, et al. Copy number variations in a cohort of 420 individuals with neurodevelopmental disorders from the south of Brazil. Sci Rep. 2019;9(1):17776. 10.1038/s41598-019-54347-z. Published 2019 Nov 28. 31780800 10.1038/s41598-019-54347-z PMC6882836

