# Supplementary material for: DNA copy number variations and craniofacial abnormalities in 1,457 children with neurodevelopmental disorders
Source: Ital J Pediatr. 2025 Jan 23;51:9. doi: 10.1186/s13052-025-01839-6 (PMC11756179; doi:10.1186/s13052-025-01839-6)
Supplement: Supplementary file 1 — Supplementary Material 1. [file 13052_2025_1839_MOESM1_ESM.docx]

**Table S1 Results of genetic testing and ratings according to ACMG and Clingene guidelines**

| **No.** | **Sex** | **CNVs type** | **Variation position** | **Total ACMG score** | **ACMG pathogenic evidence** |
| --- | --- | --- | --- | --- | --- |
| 1 | F | Del | chr4:331705-16228080:loss1 | 2.75 | 1A;2A;2G;3C;4B;4C;4E |
| 2 | F | Del | chr15:22892176-28566579:loss1 | 2.05 | 1A;2A;2G;3A;4B;4C;4E |
| 3 | M | Del | chr17:29298092-30326022:loss1 | 1.75 | 1A;2A;2G;3A;4B;4C;4E |
| 4 | M | Del | chr22:51063572-51169740:loss1 | 1.65 | 1A;2C-1;3A;4B;4E |
| 5 | M | Del | chr8:1004-79794 | 5 | PVS1-M,PM3,PM2-supporting |
| 6 | F | Del | chr5:92294-19839095:loss1 | 2.95 | 1A;2A;2G;3C;4B;4C;4E |
| 7 | F | Del | chrX:1-155270560:loss1 | 2.4 | 1A;2A;2G;3C;4C;4E |
| 8 | F | Del | chrX:1-155270560:loss1 | 2.4 | 1A;2A;2G;3C;4C;4E |
| 9 | M | Del | chr7:98983338-100239132:loss1 | 2 | 1A;2A;2G;3C;4E |
| 10 | M | Del | chr22:18900688-21351637:loss1 | 2.95 | 1A;2A;2G;3C;4B;4C;4E |
| 11 | M | Del | chr7:98983338-100860555:loss1 | 2 | 1A;2A;2G;3C;4E |
| 12 | M | Del | chr15:31196867-32404100:loss1 | 1.75 | 1A;2A;2G;3A;4B;4C;4E |
| 13 | F | Del | chrX:1-155270560:loss1 | 2.4 | 1A;2A;2G;3C;4C;4E |
| 14 | F | Del | chr22:50297486-51178405:loss1 | 2.35 | 1A;2A;2G;3B;4B;4C;4E |
| 15 | F | Del | chr22:50832337-51220722:loss1 | 1.75 | 1A;2A;2G;3A;4B;4C;4E |
| 16 | M | Del | chr7:72717592-74251505:loss1 | 2.5 | 1A;2A;2G;3B;4B;4C;4E |
| 17 | M | Del | chr15:23605426-28632839:loss1 | 2.05 | 1A;2A;2G;3A;4B;4C;4E |
| 18 | M | Del | chr13:30341391-43987050:loss1 | 2.15 | 1A;2A;2G;3C;4E |
| 19 | M | Del | chr13:35883664-61141775:loss1 | 2.65 | 1A;2A;2G;3C;4B;4E |
| 20 | M | Del | chr3:195686791-197273348:loss1 | 1.9 | 1A;2A;2G;3A;4B;4C;4E |
| 21 | M | Del | chr7:7025 1021  c.2305G>A(p.V769I | 1.65 | 1A;2A;2G;3A;4B;4E |
| 22 | F | Del | chr17:29298092-30326022:loss1 | 1.75 | 1A;2A;2G;3A;4B;4C;4E |
| 23 | F | Del | chr22:50297486-51183611:loss1 | 2.35 | 1A;2A;2G;3B;4B;4C;4E |
| 24 | M | Del | chrX:154719776-154842597:loss1 | 0 | 1A;2G;3A |
| 25 | M | Del | chr7:72716254-74417830:loss1 | 2.5 | 1A;2A;2G;3B;4B;4C;4E |
| 26 | F | Del | chr7:72657706-74161386:loss1 | 1.45 | 1A;2A;3B |
| 27 | M | Del | chr16:29556331-30199917:loss1 | 2.8 | 1A;2A;2G;3B;4B;4C;4E;4G |
| 28 | M | Del | chrX:23018154-23412322:loss1 | 1.15 | 1A;2C-1;3A;4B |
| 29 | F | Del | Xp22.33p11.21 | 1.8 | 1A;2A;2G;3A;4E |
| 30 | F | Del | chr17:14095286-15472395:loss1 | 1.6 | 1A;2A;2G;3A;4C;4E |
| 31 | M | Del | chr3:150340152-156570841:loss1 | 1.45 | 1A;2A;2G;3B |
| 32 | M | Del | chr16:3900297-4245787:loss1 | 1 | 1A;2C-1;2G;3A |
| 33 | M | Del | chr16:15125572-16415108:loss1 | 1.9 | 1A;2A;2G;3A;4B;4C;4E |
| 34 | M | Del | chr3:195686791-197282952:loss1 | 1.9 | 1A;2A;2G;3A;4B;4C;4E |
| 35 | F | Del | chr5:92294-12575172:loss1 | 2.95 | 1A;2A;2G;3C;4B;4C;4E |
| 36 | M | Del | chr5:133292540-134785657:loss1 | 0.9 | 1A;3A;4B |
| 37 | M | Del | chr9:116799-6015649:loss1 | 2.05 | 1A;2A;2G;3B;4C;4E |
| 38 | M | Del | chr15:20739496-32786302:loss1 | 1.75 | 1A;2A;2G;3B;4E |
| 39 | F | Dell | chrX:200843-52118798:loss1 | 2.5 | 1A;2A;2G;3C;4A;4E |
| 40 | F | Del | chr16:29802081-30102514:loss1 | 1.4 | 1A;2A;3A;4C;4E |
| 41 | M | Del | chr7:72717394-74133304:loss1 | 2.05 | 1A;2A;2G;3A;4B;4C;4E |
| 42 | F | Del | chr7:72688002-74175900:loss1 | 2.05 | 1A;2A;2G;3A;4B;4C;4E |
| 43 | M | Del | chr7:72825920-74180952:loss1 | 2.05 | 1A;2A;2G;3A;4B;4C;4E |
| 44 | M | Del | chr7:72693718-74164952:loss1 | 2.05 | 1A;2A;2G;3A;4B;4C;4E |
| 45 | F | Del | chr7:72798515-75451214:loss1 | 2.05 | 1A;2A;2G;3B;4B;4E |
| 46 | F | Del | chr7:72742276-74133260:loss1 | 2.05 | 1A;2A;2G;3A;4B;4C;4E |
| 47 | F | Del | chr22:18894077-21652015:loss1 | 2.95 | 1A;2A;2G;3C;4B;4C;4E |
| 48 | M | Del | chr7:72717592-74151025:loss1 | 2.05 | 1A;2A;2G;3A;4B;4C;4E |
| 49 | M | Del | chr7:72741491-74035247:loss1 | 2.05 | 1A;2A;2G;3A;4B;4C;4E |
| 50 | M | Del | chr7:72689356-75099853:loss1 | 1.9 | 1A;2A;3C |
| 51 | F | Del | chr7:72700490-74223715:loss1 | 2.5 | 1A;2A;2G;3B;4B;4C;4E |
| 52 | F | Del | chr7:72583913-74166348:loss1 | 2.05 | 1A;2A;2G;3A;4B;4C;4E |
| 53 | F | Del | chr7:72717394-74133304:loss1 | 2.05 | 1A;2A;2G;3A;4B;4C;4E |
| 54 | F | Del | chr7:72700231-75233665:loss1 | 2.05 | 1A;2A;2G;3B;4B;4E |
| 55 | F | Del | chr17:29111204-30380347:loss1 | 1.75 | 1A;2A;2G;3A;4B;4C;4E |
| 56 | F | Del | chr10:81697607-89127185:loss1 | 2.5 | 1A;2A;2G;3B;4B;4C;4E |
| 57 | M | Del | chr16:14858146-18569143:loss1 | 1.5 | 1A;2A;2G;3A;4C;4E |
| 58 | M | Del | chr14:55408207-58070880:loss1 | 1 | 1A;2A;2G;3A |
| 59 | M | Del | chr1:179782204-193221391:loss1 | 2.1 | 1A;2A;2G;3C;4E |
| 60 | F | Del | chr16:29571432-30218221:loss1 | 2.8 | 1A;2A;2G;3B;4B;4C;4E;4G |
| 61 | F | Del | chr14:55462320-58915287:loss1 | 1 | 1A;2A;2G;3A |
| 62 | F | Del | chr22:18893867-21414817:loss1 | 2.95 | 1A;2A;2G;3C;4B;4C;4E |
| 63 | F | Del | chrX:200843-7268972:loss1 | 1.35 | 1A;2C-1;2G;3B |
| 64 | M | Del | chr15:98982844-102462794:loss1 | 1.95 | 1A;2A;2G;3A;4B;4C;4E |
| 65 | F | Del | 45,X | 2.4 | 1A;2A;2G;3C;4C;4E |
| 66 | F | Dup | chrX:102564517-102983131:gain1 | 0 | 1A;3A |
| 67 | F | Dup | chr15:22082360-32455556:gain1 | 1.75 | 1A;2A;2G;2H;3A;4B;4C;4E |
| 68 | M | Dup | chr16:29454225-30256909:gain1 | 2.05 | 1A;2A;2G;2H;3B;4C;4E |
| 69 | M | Dup | chr15:22833394-28096702:gain1 | 1 | 1A,2A,2H,3A |
| 70 | F | Dup | chr15:20739496-34828164:gain1 | 1.85 | 1A;2A;2G;2H;3B;4C;4E |
| 71 | M | Dup | chr15:22892176-32462384:gain1 | 1.75 | 1A;2A;2G;2H;3A;4B;4C;4E |
| 72 | M | Dup | chr16:28995106-33263695:gain1 | 1.9 | 1A;2A;2G;2H;3C |
| 73 | F | Dup | chr15:23810773-28566579:gain1 | 2.5 | 1A;2A;2G;3A;4B;4C;4E |
| 74 | M | Dup | chr1:146465858-147624621:gain1 | 2.05 | 1A;2A;2G;3A;4B;4C;4E |
| 75 | F | Dup | chr22:18905828-21824110:gain1 | 2.2 | 1A;2A;2G;2H;3B;4B;4C;4E |
| 76 | M | Dup | chr5:154242838-180687814:gain1 | 2.2 | 1A;2A;2G;2H;3C;4E |
| 77 | M | Dup | chr17:14095286-15472395:gain1 | 1.7 | 1A;2A;2G;2H;3A;4B;4C;4E |
| 78 | M | Dup | chr7:72717394-74148369:gain1 | 2.05 | 1A;2A;2G;2H;3A;4B;4C;4E |
| 79 | M | Dup | chr3:195513760-197949395:gain1 | 1.2 | 1A;2A;3A;4C;4E |
| 80 | M | Dup | 47,XXY | 1.9 | 1A;2A;3C |
| 81 | F | Dup | chr1:223480001-231860000:gain1 | 2 | 1A;2A;2G;2H;3C;4E |
| 82 | M | Dup | chr22:18893867-21414817:gain1 | 2.2 | 1A;2A;2G;2H;3B;4B;4C;4E |
| 83 | F | Dup | chr15:20739496-28806215:gain1 | 2.05 | 1A;2A;2G;2H;3A;4B;4C;4E |
| 84 | M | Dup | chr5:113365-23527882:gain1 | 2.65 | 1A;2A;2G;2H;3C;4B;4C;4E |
| 85 | F | Dup | chrX:51075817-92965165:gain1 | 1.9 | 1A;2A;2G;2H;3C |
| 86 | F | Dup | 47,XXX | 1.9 | 1A;2A;3C |
| 87 | F | Dup | chr17:16748694-20363785:gain1 | 2.5 | 1A;2A;2G;2H;3B;4B;4C;4E |
| 88 | M | Dup | chr1:146035841-147907465:gain1 | 1.75 | 1A;2A;2G;3A;4B;4C;4E |
| 89 | M | Dup | chr1:211432902-249212562:gain1 | 2.3 | 1A;2A;2G;2H;3C;4C;4E |
| 90 | M | Dup | chr16:14796854-16388622:gain1 | 1.4 | 1A;2A;3A;4C;4E |
| 91 | F | Dup | chr9:132662238-141121553:gain1 | 2.5 | 1A;2A;2G;2H;3C;4B;4E |
